# Computed tomography–guided suture anchor localizer placement for multiple pulmonary nodule localization

**DOI:** 10.20452/wiitm.2025.17991

**Published:** 2025-11-06

**Authors:** Zheng Gong, Tong Zhou, Yong‑Guang Gao

**Affiliations:** Department of Cardiothoracic Surgery Xuzhou Cancer Hospital China; Department of Nuclear Medicine Xuzhou Cancer Hospital China; Department of Radiology Xuzhou Cancer Hospital China

**Keywords:** computed tomography, multiple nodules, pulmonary nodule, suture anchor localizer, video-assisted thoracoscopic surgery

## Abstract

**INTRODUCTION:**

Computed tomography (CT)-guided suture anchor localizer (SAL) placement is increasingly used to facilitate preoperative localization of pulmonary nodules (PNs) before video-assisted thoracoscopic surgery (VATS). Although this approach is well established for single nodules, evidence regarding its application in multiple nodules remains limited.

**AIM:**

We aimed to evaluate the safety and efficacy of CT-guided SAL placement for simultaneous localization of multiple PNs.

**MATERIALS AND METHODS:**

A 2-center retrospective study was conducted enrolling patients who underwent CT-guided SAL placement for multiple PNs followed by VATS resection between January 2023 and December 2024. A contemporaneous cohort undergoing single PN localization served as the control group. Clinical outcomes and procedural complications were compared between the groups.

**RESULTS:**

A total of 49 patients underwent the localization of 106 PNs in the multiple-nodule group, whereas 163 patients underwent the localization of 163 single PNs in the single-nodule group. The technical success of SAL placement was 100% in both groups. Localization time was longer in the multiple-nodule group (*P *<⁠0.001). Pneumothorax and intrapulmonary hemorrhage occurred more frequently after multiple SAL placements (36.7% and 28.6%, respectively), as compared with single-nodule localization (18.9% and 16%; *P* = 0.007 and *P* = 0.048, respectively). Despite these differences, the technical success of VATS sublobar resection was 100% in both cohorts.

**CONCLUSIONS:**

CT-guided SAL placement is a reliable and safe method for preoperative localization of multiple PNs. Our findings supportin its clinical utility in patients undergoing VATS.

## INTRODUCTION 

Pulmonary nodules (PNs) are defined as rounded or ovoid lesions within the lung parenchyma, measuring 30 mm or less in maximal diameter.^1^^,^^2^^,^^3^ Most PNs are detected incidentally on chest computed tomography (CT).^1^ While low-risk PNs are typically managed through periodic imaging surveillance, higher-risk nodules often require histologic confirmation or surgical removal.^1^ Video-assisted thoracoscopic surgery (VATS) has become the preferred minimally-invasive technique for the resection of high-risk PNs.^4^

Sublobar resection by VATS is especially advantageous for early-stage lung cancer and screening-detected nodules.^5^^,^^6^^,^^7^ Accurate preoperative localization of PNs enhances the precision and success of sublobar resections, and reduces the risk of incomplete resection or conversion to thoracotomy.^8^^,^^9^^,^^10^ This need is even more pronounced in patients with multiple high-risk PNs, in whom precise localization can minimize the extent of lung tissue removal. Suture anchor localizer (SAL; Senscure, Ningbo, China) is a novel localization device, increasingly adopted for CT--guided PN targeting.^8^^,^^9^ However, its effectiveness and safety for simultaneous localization of multiple PNs remain poorly defined.

**FIGURE 1:**
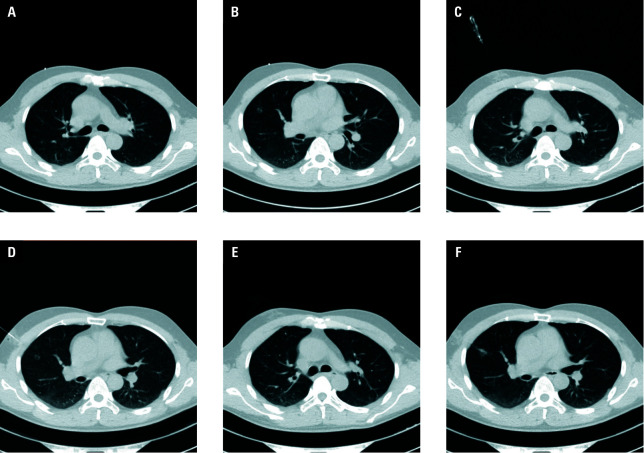
A, B - Ground-glass nodules (GGNs; arrows) located in the right upper lobe of the lungs; C, D - guiding needle puncture near the target GGN (arrows); E, F - placement of suture anchor localizers (arrows) near the GGNs for localization

## AIM 

This study was designed to evaluate the clinical performance and safety profile of CT-guided SAL placement for localizing multiple PNs prior to VATS resection.

## MATERIALS AND METHODS 

Ethics This retrospective study was approved by the Ethics Committees of the Xuzhou Cancer Hospital (2025-02-000-K03) and the Xuzhou Central Hospital (XZXY-LJ-20150110-090). Given the retrospective design, the requirement for informed consent was waived.

### Patient enrollment 

From January 2023 to December 2024, consecutive patients who underwent CT--guided SAL placement for localization of multiple PNs followed by VATS resection were enrolled. Inclusion criteria were: 1) presence of more than 1 high-risk PN, 2) requirement for localization of more than 1PN, 3) multiple unilateral nodules, and 4) age between 18 and 80 years. Exclusion criteria comprised: 1) nodules smaller than 6 mm , 2) typical metastatic lung nodules, and 3) contraindications to localization or VATS. A control group consisting of patients who underwent single PN localization during the same period was also enrolled.

### Computed tomography-guided localization using a suture anchor localizer 

All localization procedures were performed under CT guidance with local anesthesia. Puncture trajectories were planned based on PN location, and patient positioning was adjusted accordingly; repositioning during the procedure was permitted. Using a 20-G introducer needle,

the operator advanced the tip to within approximately 10 mm of the target PN. The SAL device was then deployed at the planned site, and the guiding needle was withdrawn. Multiple nodules were localized sequentially in a single session (FIGURE 1). After each deployment, repeat CT was performed to confirm accurate placement and detect any immediate complications.

### Video-assisted thoracoscopic surgery procedures 

VATS resections were generally performed within 3 hours of localization. Under SAL guidance, sublobar resection procedures, including wedge resections and segmentectomies, were conducted. Wedge resection was performed first, with segmentectomy reserved for cases where it failed to achieve adequate margins. The excised nodules were sent for intraoperative frozen-section analysis. If invasive lung cancer was identified, systematic lymph node dissection was performed. Lobectomy was reserved for lesions smaller than 2 cm or with a sol-id/ground-glass ratio below 50%. All target nodules were resected during the same VATS session.

### Assessment 

Technical success of localization was defined by the simultaneous fulfillment of all of the following criteria: 1) SAL was clearly visible during VATS, 2) no evidence of SAL dislodgement occurred prior to resection, and 3) the target PN was confirmed to be present within the excised lung tissue. Technical success of VATS sublobar resection was defined as the resected lung parenchyma containing the target nodule. Localization time was defined as the time from the initial to the last CT scan during the localization procedure. VATS time was defined as the time from entering to exiting the operation room. The primary end point was the localization technical success rate. Secondary end points included localization time, procedure-related complications, type of surgical resection, operative duration, intraoperative blood loss, and final pathologic diagnosis. Postoperatively, all participants underwent follow-up chest CT at 1,3 , and 6 months postsurgery, and thereafter at 6 -month intervals.

**FIGURE 2:**
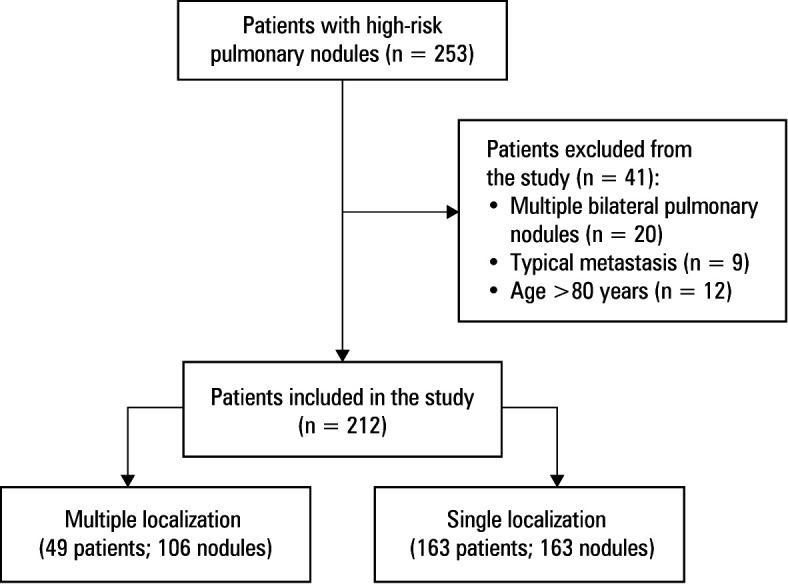
Flow chart of the study

**TABLE 1:** Baseline characteristics of the patients

Parameter		Multiple-nodule group (*n* = 49 )	Single-nodule group (*n*= 163)	*P *value
Number of PNs	2	41 (83.7)	-	-
	3	8 (16.3)	-	
Age, y , mean (SD)/coefficient of dispersion		54.4 (11.2)/0.21	55.8 (12.1)/0.22	0.52
Sex	Men	16 (32.7)	57 (35)	0.77
	Women	33 (67.3)	106 (65)	
PN diameter, mm, mean (SD)/coefficient of dispersion		7.8 (2.3)/0.29	8.7 (2.4)/0.28	0.001
PN depth, mm, median (IQR)/ coefficient of dispersion		10 (5-15)/0.76	10 (5-18)/0.8	0.11
PN nature	Solid	20 (18.9)	46 (28.2)	0.08
	GGN	86 (81.1)	117 (71.8)	
PN location	Left upper lobe	17 (16)	32 (19.6)	0.04
	Left lower lobe	10 (9.4)	34 (20.9)	
	Right upper lobe	46 (43.4)	59 (36.2)	
	Right middle lobe	5 (4.7)	11 (6.7)	
	Right lower lobe	28 (26.5)	27 (16.6)	
	All target PNs in the same lobe	23 (46.9)	-	

Data are presented as numbers (percentages) unless indicated otherwise.

Abbreviations: GGN, ground-glass nodule; IQR, interquartile range; PN, pulmonary nodule

### Statistical analysis 

Data were analyzed with SPSS Statistics software, version 16.0 (IBM, Armonk, New York, United States). Quantitative variables were expressed as mean (SD) or median (interquartile range [IQR]), depending on data distribution. Between-group comparisons were conducted using the t test or the Mann-Whitney test, as appropriate. Categorical variables were presented as counts (percentages) and evaluated with the χ2 test. Multivariable logistic regression analysis was applied to explore potential risk factors. When performing the logistic analysis, the factors with a P value below 0.1 in the univariable logistic analysis were included in the multivariable logistic analysis. A P value below 0.05 was deemed significant.

## RESULTS 

### Patient characteristics 

In the multiplenpdule group (MNG), 49 patients underwent CT--guided localization of 106 PNs , including 41 individuals with 2 nodules and 8 patients with 3 nodules. In the single-nodule group (SNG), a single PN was localized in each of the 163 participants. The flow chart of the study is presented in FIGURE 2. Baseline characteristics of the study population are summarized in TABLE 1.

Localization outcomes Technical success of SAL placement reached 100% in both groups (TABLE 2). However, mean (SD) time of localization procedures was longer for multiple nodules than single nodules (22.3 [9.7] vs 9.4 [4.1] min; P < 0.001 ). All patients in the MNG underwent single-session CT-guided localization; 27 individuals (55.1%) required intraprocedural changes in the body position. Target nodules were confined to a single lobe in 23 patients ( 46.9% ).

Complication rates were higher in the MNG, with pneumothorax and intrapulmonary hemorrhage occurring in 36.7% and 28.6% of the individuals, respectively, as compared with 18.9% and 16% in the SNG ( P = 0.007 and P = 0.048, respectively). In the MNG, 7 participants ( 14.3% ) developed pneumothorax after localization of the first nodule, whereas 11 patients ( 22.4% ) experienced pneumothorax after localization of their second or third nodule. None of the patients required chest tube drainage or experienced a delay in subsequent VATS. Logistic regression did not identify significant predictors of either pneumothorax or hemorrhage after multiple-nodule localization.

### Video-assisted thoracoscopic surgery outcomes 

As shown in TABLE 3, technical success of VATS sublobar resection was 100% in both groups. Mean (SD) blood loss was similar between the MNG and SNG ( 50[20 − 100] vs 20[10 − 50]ml; P = 0.1). Operative time was longer for the patients with multiple localizations than single-nodule cases ( 80vs70 min ; P = 0.01 ). No conversions to thoracotomy occurred. All patients in the MNG successfully underwent 1-stage resections of all targeted nodules. The types of sublobar resection were comparable between the groups ( P = 0.81 ). Ten individuals in the MNG and 22 in the SNG underwent lobectomy following sublobar resection. Pathologic diagnoses of the excised nodules are listed in table 3. Notably, 25 patients ( 51% ) in the MNG were diagnosed with synchronous multiple primary lung cancers.

**TABLE 2:** Localization-related outcomes

Parameter		Multiple-nodule group (*n* = 49)	Single-nodule group (*n* = 163)	*P *value
Technical success rate, %		100	100	-
Body position change		27 (55.1)	-	-
Localization time, min, mean (SD)/coefficient of dispersion		22.3 (9.7)/0.43	9.4 (4.1)/0.44	<0.001
Complication	Pneumothorax	18 (36.7)	30 (18.4)	0.007
	Intrapulmonary hemorrhage	14 (28.6)	26 (16)	0.048

Data are presented as numbers (percentages) unless indicated otherwise.

**TABLE 3:** Surgery-related outcomes

Parameter		Multiple-nodule group (*n* = 49 )	Single-nodule group (*n* = 163 )	*P *value
Technical success rate of sublobar resection, %		100	100	-
VATS time, min, median (IQR)/ coefficient of dispersion		80 (60-115)/0.59	70 (50-95)/0.57	0.01
Blood loss, ml, median (IQR)/ coefficient of dispersion		50 (20-100)/0.89	20 (10-50)/1.02	0.1
VATS type	Sublobar resection alone	39 (79.6)	141 (86.5)	0.32
	Sublobar resection + lobectomy	10 (9.4)	22 (13.5)	
Sublobar resection type	Wedge resection	72 (67.9)	113 (69.3)	0.81
	Segmentectomy	34 (32.1)	50 (30.7)	
Pathologic diagnosis	Adenocarcinoma	73 (68.9)	132 (81)	0.09
	Squamous carcinoma	0	1 (0.6)	
	Benign neoplasm	25 (23.6)	24 (14.7)	
	Other	8 (7.5)	6 (3.7)	
Multiple lung cancer		25 (51)	-	-

Data are presented as numbers (percentages) unless indicated otherwise.

Abbreviations: VATS, video-assisted thoracoscopic surgery; others, see TABLE 1

### Follow-up 

All study participants were monitored for 6 − 30 months (median [IQR], 17 [8-22] mo). No new pulmonary nodules were identified during follow-up.

## DISCUSSION 

Approximately 20% of the patients with PNs present with multiple high-risk lesions.^11^ One-stage VATS resection has been associated with improved outcomes, as compared with staged procedures, by reducing the likelihood of disease progression.^12^ Nevertheless, complete resection of all suspicious lesions while preserving lung function remains a clinical challenge. This study demonstrated 2 principal findings: 1) CT-guided simultaneous SAL placement enables reliable localization of multiple nodules, and 2) SAL-assisted localization facilitates 1-stage VATS sublobar resection of multiple nodules with a high technical success rate.

Traditional localization methods utilizing coils, hook-wires, and injectable markers have long been used to guide multiple PN resections.^12^^,^^13^^,^^14^ However, these materials are not specifically designed for pulmonary application and may be associated with dislodgement, patient discomfort, or procedural complexity. In contrast, the SAL device offers several advantages: 1) a 4-hook anchor tip that firmly engages the lung parenchyma, 2) a flexible suture connecting the anchor to serve as a clear intraoperative marker, and 3) a softer design to minimize patient discomfort.

Evidence suggests that sublobar resection is an effective standard approach for small (  ≤ 2 cm ), peripherally located lung cancers.^5^^,^^6^^,^^7^ Fan et al^15^ have argued that lobectomy should be reserved for invasive tumors with a solid/ground-glass ratio exceeding 50%. Consequently, multiple sublobar resections represent a rational strategy for patients with multiple nodules, maximizing parenchymal preservation and lung function. Preoperative localization helps avoid unnecessary lobectomy and ensure precise margins. In this study, although the time required for CT-guided localization increased proportionally to the number of nodules, technical success rate remained uniformly high at 100% for both single and multiple localizations. This underscores the feasibility of SAL for managing multiple nodules in a single session. Furthermore, complication rates, though they were higher in the cases with multiple placements, remained clinically manageable, with no need for chest tube placement or procedure postponement.

Localization-related complications occurred more frequently among the patients undergoing multiple-nodule localization than those in the SNG. This observation is not unexpected, as targeting several nodules necessitates multiple puncture tracts, thereby increasing procedural complexity and tissue disruption. Nevertheless, consistent with earlier reports, such complications rarely exert a clinically meaningful impact on the subsequent VATS procedure.^16^ Im portantly, no individual in our cohort required chest tube drainage. Even in situations where chest tube placement might have become necessary, prior literature suggests that patients generally remain clinically stable and can still undergo VATS with the tube in situ and supplemental oxygen as needed.^16^

The incidence of pneumothorax in the MNG was 36.7%, falling within the range documented in previous investigations of multiple PN localization ( 21.8% − 56.8% ).^13^^,^^17^^,^^18^ Prior studies have proposed that changes in patient positioning during needle placement could influence pneumothorax risk.^18^^,^^19^ However, the present analysis did not identify any independent risk factors for pneumothorax. This absence of association may stem from the limited sample size of our study.

Similarly, the rate of intrapulmonary hemorrhage observed in the MNG ( 28.6% ) was comparable to previously reported figures (18.9% − 27.5%).^13^^, ^^17^^,^^18^

In accordance with the surgical criteria employed for this study, lobectomy was reserved for the patients with invasive lung cancer exceeding 2 cm in diameter or demonstrating a solid--to-ground glass ratio greater than 50%. The proportion of patients undergoing lobectomy was 9.4% in the MNG and 13.5% in the SNG-values broadly in line with previously reported rates for multiple-nodule resections (8.9% and 7.7%). 18 Notably, no patient in either cohort developed new pulmonary nodules during follow-up. Collectively, these outcomes underscore the appropriateness and effectiveness of this VATS-based treatment strategy.

Despite the encouraging results, several limitations of this analysis should be acknowledged. First, its retrospective design inherently introduced selection bias and restricted causal inference. Second, this study employed exclusively the SAL device, thereby precluding direct comparisons with other localization techniques, such as coils, hook--wires, or liquid markers. Third, although conducted at 2 centers to enhance generalizability, the involvement of multiple operators with varying levels of experience may have contributed to outcome variability and additional bias.

## CONCLUSIONS 

In summary, these findings indicate that CT-guided SAL placement is a reliable, safe, and effective approach for preoperative localization of multiple PNs.

## JOURNAL INFORMATION

Videosurgery and Other Miniinvasive Techniques is an official journal of the Videosurgery Foundation.
